# Neuroinvasive Free-Living Amoebae Pathogenesis, Neuroinflammation and Therapeutic Challenges

**DOI:** 10.3390/ijms27136056

**Published:** 2026-07-06

**Authors:** Oliwia Pawelec-Pęciak, Karolina Kot, Danuta Kosik-Bogacka, Natalia Łanocha-Arendarczyk

**Affiliations:** Department of Biology, Parasitology, and Pharmaceutical Botany, Pomeranian Medical University in Szczecin, Powstańców Wielkopolskich 72, 70-111 Szczecin, Poland; oliwia.pawelec.peciak@pum.edu.pl (O.P.-P.); karolina.kot@pum.edu.pl (K.K.); danuta.kosik.bogacka@pum.edu.pl (D.K.-B.)

**Keywords:** *Naegleria fowleri*, *Acanthamoeba* spp., pathogenesis, neuroinflammation, immune responses, virulence mechanisms, diagnostic and therapeutic challenges

## Abstract

Neuroinvasive free-living amoebae (FLA), particularly *Naegleria fowleri* and *Acanthamoeba* spp., are responsible for rare but devastating infections of the central nervous system (CNS). Approximately 480 cases of primary amoebic meningoencephalitis (PAM) and fewer than 200 well-documented cases of *Acanthamoeba*-associated granulomatous amoebic encephalitis (GAE) have been reported worldwide. Mortality rates frequently exceed 90%. PAM typically develops following exposure to warm freshwater contaminated with *N. fowleri* and progresses rapidly in otherwise healthy individuals. In contrast, GAE usually follows a more indolent course and occurs predominantly in immunocompromised hosts. Despite their distinct clinical courses, both infections are characterized by CNS invasion, amoeba-mediated tissue destruction, blood–brain barrier (BBB) disruption, and host inflammatory responses. These processes drive neuroinflammation, neuronal injury, and neurological deterioration. Early diagnosis remains challenging because clinical manifestations are nonspecific and disease progression can be either fulminant or initially subtle. Therapeutic management is hindered by poor CNS drug penetration, limited efficacy of currently available therapies, treatment-related toxicity, and the absence of standardized treatment protocols or controlled clinical trials. This narrative review critically synthesizes current evidence on CNS invasion, neuroinflammation, neuropathology, diagnostic challenges, and therapeutic strategies in neuroinvasive FLA infections. It also highlights key translational priorities, including earlier diagnosis, standardized treatment protocols, stronger clinical evidence, and improved CNS-targeted drug delivery.

## 1. Introduction

Neuropathogenic protozoa possess diverse mechanisms that enable penetration of the blood–brain barrier (BBB) and subsequent colonization of the central nervous system (CNS) [1]. Although the CNS has long been considered an immunologically privileged site, it remains vulnerable to parasite-induced inflammatory responses that disrupt neuronal homeostasis [2]. Free-living amoebae (FLA) are ubiquitous environmental protozoa capable of persisting in diverse aquatic, terrestrial, and human-associated environments [1,2,3,4,5]. Some amphizoic species can cause opportunistic human infections. Among them, *Naegleria fowleri* and *Acanthamoeba* spp. are the most clinically significant neuroinvasive FLA, causing primary amoebic meningoencephalitis and granulomatous amoebic encephalitis, respectively [2,3,4,5,6,7]. Neuroinvasive FLA infections are rare, globally reported, and associated with exceptionally high mortality. To date, approximately 480 cases of PAM and fewer than 200 well-documented cases of *Acanthamoeba*-associated GAE have been described worldwide, with case fatality rates frequently exceeding 90% [7,8,9,10,11,12]. Despite their distinct clinical courses, both infections involve CNS invasion, amoeba-mediated tissue destruction, BBB disruption, and host inflammatory responses that contribute to neuroinflammation, neuronal injury, and neurological deterioration [2,3]. In this review, neuropathogenesis refers to the processes through which FLA invade the CNS, interact with neural and immune cells, and induce tissue injury and pathology [13]. Neuroinflammation refers to an inflammatory response within the CNS triggered by infection or tissue injury, involving activation of microglia and astrocytes and the release of immune mediators [14]. In *Acanthamoeba* infection, this response may also include granulomatous inflammation, defined as organized aggregates of macrophages, lymphocytes, and other immune cells surrounding persistent pathogens or damaged tissue [7,8]. Despite decades of research, many aspects of CNS invasion, immune dysregulation, inflammatory injury, and tissue damage in FLA infections remain incompletely understood. This knowledge gap has direct clinical relevance because early diagnosis is often hindered by nonspecific manifestations, whereas effective treatment is limited by poor CNS drug penetration, treatment-related toxicity, and the lack of controlled clinical trials [7,9,10].

This narrative review examines current evidence on host–pathogen interactions, neuroinflammatory mechanisms, and neuropathological outcomes in neuroinvasive FLA infections and discusses their implications for diagnosis and treatment. In addition, it highlights key translational priorities, including earlier diagnosis, standardized treatment protocols, stronger clinical evidence, and improved CNS-directed drug delivery.

Literature Search Strategy

This manuscript was developed as a narrative, evidence-based review integrating clinical, experimental, and translational literature on neuroinvasive infections caused by FLA. Relevant publications were identified through searches of PubMed, Scopus, and Web of Science, covering studies published up to early 2026. The analysis included clinical case reports, experimental in vitro and in vivo studies, recent reviews, and available meta-analyses, with emphasis on neuropathogenesis, neuroinflammation, neuropathology, diagnosis, and treatment. Seminal earlier publications were also included when they provided foundational insights into the field.

## 2. *Naegleria fowleri* as the Causative Agent of Primary Amoebic Meningoencephalitis 

Of the more than 45 described *Naegleria* species, only *Naegleria fowleri* is recognized as a human pathogen capable of infecting both humans and several animal species [9]. This thermophilic free-living amoeba occurs predominantly in warm freshwater environments, including lakes, rivers, thermal waters, and inadequately disinfected recreational water systems, particularly during warmer months of the year [10,11]. For PAM, the estimated risk following recreational freshwater exposure is extremely low, at approximately one case per 2.5 million exposed swimmers [11,12].

Clinically, PAM represents a medical emergency because of its abrupt onset, aggressive CNS invasion, and rapid progression to life-threatening neurological failure [12,15]. Most published reports originate from the United States, Central America, Australia, and several Asia countries, although cases have also been documented in Europe [5,16]. One of the best-documented European outbreaks occurred in Ústí nad Labem in the former Czechoslovakia, where fatal PAM cases were linked to exposure to a heated indoor swimming pool [17]. The expanding geographic distribution of *N. fowleri* is influenced by both environmental and water-system conditions. Climate warming and thermal pollution may expand suitable habitats for this thermophilic amoeba by increasing surface-water temperatures [18]. Impaired water quality, including low disinfectant residuals, turbidity, biofilm formation, and microbial or algal overgrowth, may further facilitate its persistence in natural and engineered aquatic systems [19]. This risk is illustrated by a PAM case linked to an artificial whitewater river, where warm, turbid water, low chlorine concentrations, and extensive algal growth favored *N. fowleri* persistence [20]. These observations support the inclusion of environmental and water-quality parameters in assessments of FLA transmission risk. Certain cultural or religious practices involving nasal exposure to untreated water may also increase the risk of PAM in endemic regions or settings characterized by high ambient temperatures [21]. Considering the thermophilic nature of *N. fowleri*, the relatively low number of reported cases in tropical regions likely reflects substantial underdiagnosis.

The life cycle of *N. fowleri* comprises three morphological stages: the trophozoite (amoeboid form, 7–35 μm), the flagellate (10–16 μm), and the cyst (7–10 μm) [22]. The trophozoite is the invasive and proliferative stage responsible for human infection, whereas the cyst is an environmentally resistant form that enables survival under unfavorable conditions. Importantly, only trophozoites are observed in human brain tissue during PAM, as cyst formation does not occur within the CNS [22,23].

### 2.1. Transmission to the Host and Clinical Manifestations of PAM

PAM typically results from nasal exposure to water contaminated with *N. fowleri* trophozoites, particularly during recreational or domestic contact with warm freshwater [24,25]. Young males are disproportionately affected, most likely because of greater exposure to aquatic environments and potential age-related anatomical susceptibility of the olfactory route [15,26]. After entering the nasal cavity, trophozoites attach to the olfactory epithelium and migrate through the cribriform plate to the olfactory bulbs, thereby gaining direct access to the brain [5,24,27]. Clinical symptoms usually develop within 1–7 days and initially include headache, fever, nausea, fatigue, and occasionally anosmia. As CNS invasion progresses, patients may develop neck stiffness, seizures, confusion, photophobia, hallucinations, personality changes, coma, and signs of increased intracranial pressure [24,28]. Early nonspecific manifestations often resemble bacterial or viral meningitis, which may delay timely diagnosis and therapeutic intervention [9]. PAM typically progresses rapidly, with death occurring within 1–2 weeks of symptom onset. Consequently, many cases are diagnosed only post-mortem [5].

### 2.2. Macroscopic and Microscopic Findings in PAM

**Figure 1:**
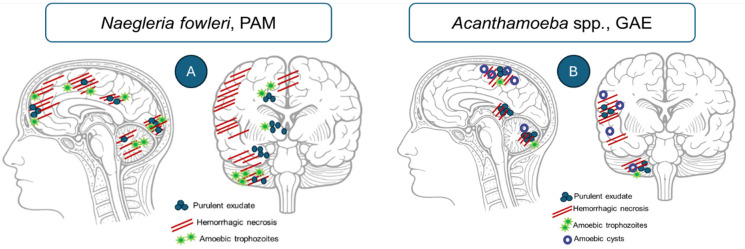
Representative histopathological alterations in brain sections. (**A**) Histopathological changes characteristic of primary amoebic meningoencephalitis (PAM). (**B**) Histopathological changes characteristic of granulomatous amoebic encephalitis (GAE). (The figure was prepared by the authors using Microsoft PowerPoint LTSC MSO 2021 (Version 16.0.14334.20756, 64-bit; Microsoft Corporation). Only the graphical brain illustration was generated with the assistance of ChatGPT (OpenAI); the scientific content, labels, and interpretation were prepared and verified by the authors).

The neuropathology of PAM reflects rapid amoebic invasion through the olfactory pathway, and extensive tissue destruction within the anterior regions of the brain. Macroscopically, the brain is typically swollen and edematous, with evidence of increased intracranial pressure and, in severe cases, herniation of the uncus or cerebellar tonsils (Figure 1). The most characteristic lesions include hemorrhage, necrosis, and inflammation involving the olfactory bulbs, olfactory tracts, frontal cortex, and adjacent frontal and temporal regions [11,29,30]. The cerebral hemispheres may appear soft, hyperemic, and congested, and hemorrhagic exudate may be present along the meningeal surfaces [5,11]. Microscopically, PAM is characterized by acute necrotizing and hemorrhagic meningoencephalitis, with extensive destruction of cortical gray matter and inflammatory involvement of the leptomeninges [31]. Tissue damage may affect multiple CNS regions, including the cerebral hemispheres, brainstem, cerebellum, and upper spinal cord [31]. Motile *N. fowleri* trophozoites can be detected in cerebrospinal fluid (CSF) or brain tissue, whereas cysts are not observed in human brain tissue during PAM [32,33]. Experimental models have similarly demonstrated extensive hemorrhagic injury and necrotic changes, particularly within the olfactory bulbs, olfactory nerves, and cerebellum [34,35].

### 2.3. Virulence Mechanisms of Naegleria fowleri

#### 2.3.1. *Adhesion and Phagocytosis*

*Naegleria fowleri* deploys a broad repertoire of virulence mechanisms that enable CNS access, tissue invasion, and rapid cerebral damage [7]. These mechanisms include contact-dependent processes, such as adhesion and phagocytosis, as well as contact-independent mechanisms, including protease secretion, extracellular vesicle release, and cytolytic activity [5,36]. Together, they support a stepwise pathogenic sequence involving attachment to host tissues, invasion of anatomical barriers, tissue destruction, and adaptation to host-derived stress signals. *N. fowleri* trophozoites exhibit high-affinity binding to extracellular matrix (ECM) components, including laminin-1, fibronectin, and type I collagen [37]. This adhesive capacity is facilitated by specialized cytoskeletal structures, notably lamellipodia, which increase the contact surface area, and focal adhesion-like complexes that mediate stable interactions with the ECM [38]. In a murine model, a ~72 kDa surface protein that interacts with the nasal epithelium has been identified. Immunofluorescence studies localized this protein to the trophozoite membrane and pseudopodia, supporting its role in host cell adhesion [39].

Adhesion is further enhanced by cathepsin B and *N. fowleri* adhesion protein 1 (Nfa1), both of which are localized to pseudopodia and specialized feeding structures termed amoebastomes (“food cups”), where they facilitate attachment, invasion, and nutrient acquisition [40,41]. Nfa1 represents a key virulence determinant through its roles in adhesion, phagocytosis, and amoebastome formation. Moreover, Nfa1 interacts with cytoskeletal and stress-response proteins, including heat shock protein 70 (Hsp70), actin (Nf-actin), and cathepsin-like proteases, thereby enhancing pathogenicity [41]. Cathepsin B contributes to host tissue adherence and may directly mediate neural tissue damage [42]. Functional inhibition of cathepsin B has been associated with improved host survival, whereas anti-Nfa1 antibodies reduce *N. fowleri*-induced cytotoxicity, supporting their relevance as promising preclinical targets for therapeutic or immunological intervention [40,42,43].

#### 2.3.2. *Invasion and Tissue Penetration*

Following these adhesion events, the parasite begins to interact more extensively with host tissues, initiating invasion processes that rely on both mechanical penetration and enzymatic activity [25]. Importantly, this invasive process is not restricted to phagocytosis. Experimental studies indicate that *N. fowleri* can compromise epithelial barrier integrity by disrupting tight-junction organization and increasing paracellular permeability, thereby facilitating penetration through the olfactory mucosa. This route of invasion is supported by amoeboid motility, cytoskeletal remodeling, adhesion to extracellular matrix components, and protease-mediated degradation of junctional and structural proteins [25,40,44]. In addition to direct host–parasite interactions, *N. fowleri* employs multiple contact-independent mechanisms that promote tissue degradation, facilitate traversal of anatomical barriers, and induce distal cytotoxic effects [5]. The amoeba secretes a variety of proteolytic enzymes, including matrix metalloproteinases-2 and -9 (MMP-2 and MMP-9, respectively), which degrade extracellular matrix components and facilitate disruption of the nasal mucosa, promoting progression along the olfactory route toward the CNS [40,45,46]. Collectively, these proteases promote extracellular matrix degradation, compromise nasal mucosal integrity, and facilitate penetration through the cribriform plate, thereby enabling CNS invasion [40,46]. Once epithelial barriers are breached, additional enzymatic mechanisms contribute to deeper tissue injury. Cysteine proteases may disrupt tight-junction integrity in BBB-associated endothelial cells, alter cytoskeletal organization, modulate the actin cytoskeleton, and facilitate trophozoite transmigration into the CNS [7,10,47]. Additional enzymatic activities, including hydrolases, phospholipases, neuraminidases, and phosphatases, contribute to host cell destruction and neural tissue damage and have also been implicated in demyelination [40]. Pore-forming polypeptides constitute another class of virulence factors that induce lysis of nucleated host cells through membrane disruption [48]. Together, these mechanisms indicate that *N. fowleri* tissue penetration is a coordinated process involving adhesion, amoeboid migration, paracellular barrier disruption, enzymatic extracellular matrix remodeling, and direct cytolytic injury [44].

#### 2.3.3. *Extracellular Vesicles, Cytotoxicity, and Stress Adaptation*

In addition to tissue destruction and invasion, immune evasion represents an important component of *N. fowleri* pathogenicity, enabling survival within the host and facilitating disease progression. Increasing attention has also focused on extracellular vesicles (EVs) released by *N. fowleri*, which constitute an important component of contact-independent pathogenicity. These vesicles exhibit hemolytic activity against erythrocytes, possess proteolytic activity, induce necrosis, and increase paracellular ion permeability, as demonstrated in Madin–Darby canine kidney (MDCK) cell models [49]. EVs are enriched in hydrolytic enzymes and cytoskeletal proteins, facilitating tissue degradation, promoting adhesion and migration, and mediating intercellular communication between amoebae and host cells [50]. In parallel, Nf23 has emerged as another virulence-associated molecule. Its expression increases after brain invasion in vivo, suggesting infection-dependent regulation and a role in disease progression [51]. Neutralization with anti-Nf23 antibodies reduces amoeba-induced cytotoxicity, further supporting its contribution to virulence [51]. Finally, although increased nitric oxide (NO) production is observed within the trophozoite microenvironment during infection, *N. fowleri* demonstrates relative resistance to NO-mediated cytotoxicity. This adaptation likely promotes parasite survival under oxidative stress and helps sustain pathogenic potential [5].

Overall, the pathogenicity of *N. fowleri* reflects the coordinated action of adhesion, phagocytosis, proteolytic degradation, barrier disruption, EV-mediated cytotoxicity, immune evasion, and resistance to host-derived stress signals. Among these mechanisms, cathepsin B, Nfa1, Nf23, and EV-associated pathways appear to be particularly promising preclinical targets for future therapeutic or immunological intervention. Collectively, these virulence factors accelerate olfactory migration, CNS invasion, edema formation, hemorrhage, necrosis, and intracranial pressure. This multifactorial pathogenic strategy helps explain the fulminant course of PAM and the narrow window available for diagnosis and treatment.

### 2.4. Immune Response to Naegleria fowleri

Exposure of the nasal cavity to *N. fowleri* trophozoites initiates a rapid mucosal immune response, although several stages of this process remain incompletely understood. After nasal entry, the amoeba must overcome physical barriers, including mucus and epithelial tight junctions [7,16]. Disruption of tight-junction proteins facilitates epithelial penetration and represents an early step in tissue invasion [52].

The first immunological barrier is mediated by respiratory antibodies, including immunoglobulin A (IgA), immunoglobulin M (IgM), and immunoglobulin G (IgG), which may reduce amoebic motility, inhibit epithelial adhesion, and promote clearance from the upper airways [53]. Experimental studies further support the protective role of antibody-mediated immunity. In a murine model of nasal infection, Fc gamma receptor III (FcγRIII) activation was associated with enhanced neutrophil-mediated amoebic clearance, reduced parasite migration, and lower risk of PAM [54]. Natural IgG and IgA antibodies against *N. fowleri* antigens have also been detected in residents of endemic areas, suggesting prior environmental exposure and potential relevance for serological diagnostics and preventive strategies [55]. Rodríguez-Mera et al. [55] further demonstrated that these antibodies recognize structurally distinct antigens of *N. fowleri* located within the cell membrane, pseudopodia, food cups, and extracellular vesicles. The immunogenicity of these antigens highlights their potential utility in serological diagnostics and, potentially, vaccine development for endemic regions.

Although antibodies may promote complement activation, *N. fowleri* exhibit resistance to complement-mediated lysis and can secrete proteases that degrade immunoglobulins, representing an important mechanism of immune evasion [7,56]. When antibody-mediated responses are insufficient, a rapid innate immune response develops, characterized by early recruitment of neutrophils to the olfactory epithelium [52,53]. Neutrophils respond through the production of reactive oxygen species (ROS), NO, and neutrophil extracellular traps. If trophozoites penetrate deeper tissues, macrophages and monocytes are also activated. Nevertheless, some amoebae evade local immune clearance, reach the lamina propria, and migrate along olfactory nerve bundles toward the brain, where neuronal pathways may partially shield them from immune effector mechanisms [53].

Once within the CNS, infection triggers a fulminant inflammatory response characterized by infiltration of neutrophils, monocytes, and eosinophils, together with activation of microglia and astrocytes [40]. At this stage, neurological injury is shaped not only by direct amoebic cytotoxicity but by a rapidly escalating pathogenic circuit in which parasite-mediated tissue destruction and host-driven immunopathology reinforce one another [5,40]. Neutrophil-rich inflammation, microglial activation, astrocyte reactivity, cytokine and chemokine release, oxidative stress, and inflammasome activation collectively amplify BBB disruption, cerebral edema, hemorrhagic–necrotic tissue injury, and increased intracranial pressure [57]. Thus, PAM represents a paradigmatic example of fulminant innate neuroinflammation, in which antimicrobial defense mechanisms become insufficient to restrict parasite invasion while simultaneously accelerating irreversible CNS damage. This response can escalate into severe neuroinflammation and widespread tissue destruction. Microglia stimulated by *N. fowleri*-derived extracellular vesicles adopt a pro-inflammatory phenotype and produce cytokines such as interleukin-6 (IL-6), interleukin-1β (IL-1β), interleukin-23 (IL-23), and tumor necrosis factor- α (TNF-α), as well as ROS and NO [58]. Interleukin-10 (IL-10) production has also been observed, suggesting an attempted regulatory response that is usually insufficient to prevent immunopathology [59]. Activated astrocytes further amplify inflammation by releasing cytokines and chemokines involved in immune-cell recruitment [60,61].

At the molecular level, amoebic trophozoites can induce ROS production, which promotes epidermal growth factor receptor (EGFR)-dependent interleukin-8 (IL-8) upregulation and EGFR-independent IL-1β expression [62]. ROS may also contribute to NLR family pyrin domain-containing 3 (NLRP3) inflammasome activation, leading to caspase-1 activation and maturation of IL-1β [62,63]. In addition, *N. fowleri* has been shown to induce ROS-dependent necroptosis in Jurkat cells, suggesting that inflammatory cell death may contribute to irreversible CNS damage [64].

Overall, the immune response to *N. fowleri* has a dual role. Early mucosal and innate responses may limit parasite migration and dissemination, whereas excessive CNS inflammation contributes to edema, tissue destruction, and irreversible neurological injury. Effective control of infection therefore likely depends on a delicate balance between protective immunity and immunopathology.

## 3. *Acanthamoeba* spp. as Etiological Agents of Granulomatous Amoebic Encephalitis 

*Acanthamoeba* spp. are globally distributed FLA that persist in diverse environmental and human-associated habitats, including aquatic systems, soil, tap water, recreational waters, medical devices, air-conditioning systems, and contact lenses [65,66,67,68,69]. Widespread human exposure is reflected by the detection of anti-*Acanthamoeba* IgG antibodies in up to approximately 80% of healthy individuals [70]. To date, at least 24 species and approximately 23 genotypes (T1–T23), defined by 18S rDNA sequencing, have been described. Genotype T4 is most frequently associated with human pathogenicity, although T1, T2, T4, T5, T10, T12, and T18 have also been implicated in GAE [71,72,73]. The clinical burden of GAE remains difficult to define. Since the first reported case in the United States in the 1970s, sporadic cases have been documented worldwide. Between 1990 and 2026, 81 cases were identified in the PubMed-indexed literature, whereas U.S. surveillance data documented 122 cases by 2020, including 20 confirmed post-mortem [74,75,76,77,78]. This discrepancy suggests substantial underestimation of GAE in the published literature and likely reflects diagnostic challenges, underreporting, and post-mortem recognition. Unlike PAM, GAE is usually reported as sporadic cases rather than outbreak-associated infection; therefore, this section focuses on representative epidemiological patterns rather than individual case descriptions.

Unlike *N. fowleri*, *Acanthamoeba* spp. have two life-cycle stages: a motile trophozoite (25–40 μm) and a resistant cyst (13–20 μm), and they lack a flagellated stage [71]. Trophozoites are metabolically active forms characterized by pseudopodia and acanthopodia, whereas cysts possess a double-layered wall composed of an outer ectocyst and an inner endocyst. This wall, rich in cellulose and chitin, enables long-term survival under adverse environmental conditions and confers resistance to many disinfectants [71,79,80]. Such resilience promotes persistence in natural and engineered water systems as well as human-associated environments, increases opportunities for exposure, and complicates prevention and eradication strategies.

### 3.1. Transmission Routes and Clinical Manifestations of Acanthamoeba-Associated GAE

GAE occurs predominantly in immunocompromised individuals, including patients receiving immunosuppressive or anticancer therapy, transplant recipients, and individuals living with HIV [8,74,81]. However, CNS involvement has also been reported in immunocompetent hosts and, rarely, in infants [82,83,84].

Infection may occur through inhalation of amoebae into the nasal cavity or lower respiratory tract, or through entry via damaged skin exposed to contaminated water or soil [16,85]. The parasite may subsequently disseminate to the CNS through hematogenous spread from primary sites in the lungs or skin, or less commonly, through direct invasion via the olfactory epithelium [86,87,88]. Nasal exposure to untreated water, including nasal irrigation with unboiled tap water or the use of inadequately cleaned continuous positive airway pressure (CPAP) devices, has been recognized as a clinically relevant risk factor [87]. Additional routes of neuroinvasion remain less well defined. Experimental studies suggest that *Acanthamoeba* spp. may migrate between the CNS and ocular structures along neural pathways, including the optic nerve [89,90]. However, direct clinical evidence supporting cornea-to-brain migration is currently lacking. Therefore, ocular-to-CNS spread should be regarded as a biologically plausible but unproven mechanism rather than an established route of GAE.

The initial manifestations of GAE are nonspecific and may mimic bacterial or viral meningoencephalitis [91]. Patients commonly present with headache, low-grade fever, nausea, dizziness, irritability, or subtle behavioral changes [92]. The disease usually follows a subacute to chronic course, with an incubation period ranging from several weeks to several months [93,94]. As CNS dissemination progresses, neurological symptoms develop in most patients and may include altered mental status, seizures, focal neurological deficits, aphasia, ataxia, diplopia, hallucinations, or meningeal signs [95,96,97,98,99]. Progressive neurological deterioration may ultimately lead to death, most often as a consequence of increased intracranial pressure [92,98]. The evolution of these clinical manifestations parallels the development of characteristic neuropathological changes within the CNS, which ultimately drive neurological deterioration and diseases progression.

### 3.2. Neuropathological Features of GAE

Clinical and neuropathological studies indicate that GAE lesions most commonly involve the cerebral cortex, particularly the frontal and temporal lobes, although deep brain structures, the brainstem, cerebellum, and posterior fossa may also be affected [75,93,99,100]. The principal neuropathological findings include focal necrotic or abscess-like lesions; granulomatous inflammation; vasculitis; and, occasionally, hydrocephalus resulting from impaired cerebrospinal fluid outflow [31,75,93]. *Acanthamoeba* cysts and trophozoites are frequently located in perivascular regions and may be surrounded by macrophages, lymphocytes, and multinucleated giant cells [31,101,102]. In immunosuppressed patients, granulomas may be poorly formed or absent, whereas necrosis and vasculitis often predominate [99].

Experimental models provide additional insight into early CNS involvement. Following intranasal inoculation in mice, cerebral hyperemia, hemorrhagic lesions, and marked damage to the olfactory bulbs have been observed, supporting the relevance of the olfactory route in CNS invasion [103]. Meningeal involvement and neuronal injury have also been reported, although the translation of these findings to human GAE requires cautious interpretation [103,104].

Histopathological confirmation is rarely available during life. Consequently, diagnosis often relies on neuroimaging; CSF analysis; molecular testing; and, when feasible, brain biopsy. Neuroimaging typically demonstrates multiple well-demarcated ring-enhancing lesions with perilesional edema, while leptomeningeal enhancement may be observed when the meninges are involved [75,93,105,106,107]. Imaging findings may vary according to immune status. Immunocompetent patients may present with disseminated tumor-like or hemorrhagic lesions, whereas immunosuppressed patients may show atypical lesions mimicking stroke, neoplasia, or other CNS infections [75,93,105,106,107]. Early-stage disease may show no detectable neuroimaging abnormalities, and amoebae are often difficult to identify in CSF, even with molecular methods [93,108]. The parasite may be present only transiently or in low numbers, which explains why brain biopsy is frequently required for definitive diagnosis [108]. CSF analysis typically reveals elevated protein concentrations, hypoglycorrhachia, and predominantly lymphocytic pleocytosis, although atypical cellular profiles, including neutrophil predominance, have been reported in selected cases [82,105,106].

Overall, the clinical and neuropathological manifestations of neuroinvasive FLA infections are strongly species-dependent. *N. fowleri* typically causes an acute, fulminant meningoencephalitis characterized by rapid neurological deterioration, whereas *Acanthamoeba* spp. more commonly produce subacute or chronic granulomatous encephalitis associated with heterogeneous neurological manifestations and multifocal CNS lesions. Table 1 summarizes these species-dependent differences, including clinical presentation, neuropathological features, diagnostic challenges, and therapeutic implications.

### 3.3. Virulence Mechanisms of Acanthamoeba spp.

The virulence of *Acanthamoeba* spp. reflects a stepwise process involving adhesion to host cells, active invasion and phagocytosis, enzymatic barrier disruption of host barriers, and induction of host-cell death. Together, these mechanisms promote local tissue injury and facilitate dissemination to the CNS, particularly in immunocompromised hosts.

#### 3.3.1. Adhesion to Host Cells

The invasive potential of *Acanthamoeba* spp. is largely determined by their ability to adhere to host tissues. This process is supported by abundant acanthopodia, with pathogenic strains exhibiting substantially more surface protrusions than non-pathogenic isolates [109,110]. Adhesion is further mediated by parasite surface proteins, including the mannose-binding protein (MBP) and laminin-binding protein (LBP), which recognize host glycoproteins and extracellular matrix components [111].

MBP, a transmembrane adhesin that recognizes mannose residues on host cells, promotes attachment and activates downstream virulence pathways involving serine proteases and metalloproteinases [112]. In vitro studies demonstrate a strong correlation between MBP expression and cytopathogenicity, with highly cytopathic strains exhibiting increased MBP levels [113,114,115]. Although direct experimental evidence in GAE remains limited, MBP-mediated adhesion to endothelial cells of the BBB is considered to represent an early step in CNS invasion [109,116].

#### 3.3.2. Host Cell Invasion, Phagocytosis, Paracellular Migration and Dissemination

Following attachment, *Acanthamoeba* spp. actively invade host tissues through phagocytic and endocytic mechanisms. Amoebae initiate actin-dependent phagocytosis, accompanied by cytoskeletal remodeling regulated by tyrosine kinase and phosphatidylinositol 3-kinase (PI3K) signaling pathways [71,117]. Subsequent phagosome maturation, vesicular trafficking and lysosomal fusion enable degradation of internalized material and contribute to host-cell injury [118,119]. In addition to direct cytolysis and phagocytosis, *Acanthamoeba* spp. may promote tissue invasion through paracellular routes. This process involves disruption of intercellular junctions; increased barrier permeability; and redistribution or degradation of tight-junction proteins, including zonula occludens-1 (ZO-1) and occludin [16,44,120,121]. Together with adhesion to extracellular matrix components and protease-mediated degradation of structural proteins, these mechanisms facilitate amoebic migration across epithelial and endothelial barriers [65]. Barrier damage may also support dissemination from peripheral sites toward the CNS. This is particularly relevant in GAE, where disease progression is thought to involve tissue invasion, hematogenous dissemination and subsequent interaction with the BBB.

#### 3.3.3. Enzymatic Mechanisms of Tissue Damage

Tissue destruction during *Acanthamoeba* spp. infection is strongly driven by secreted hydrolytic enzymes that degrade host structural and barrier-associated molecules. Serine and cysteine proteases, metalloproteinases and phospholipases target extracellular matrix components, cellular membranes and junctional proteins, thereby promoting intercellular migration, barrier penetration and host-cell injury [71,109]. Serine proteases can disrupt endothelial tight-junction proteins, including occludin, claudin-1 and ZO-1, as well as structural and immune proteins such as collagen, fibronectin, fibrinogen and immunoglobulins [122,123]. This protease-dependent destabilization of tight junctions is considered a key mechanism facilitating paracellular traversal of the BBB in GAE [98]. Additional virulence factors include parasite-derived and host-induced matrix metalloproteinases, whose dysregulated activity may amplify extracellular matrix degradation, BBB dysfunction and neuroinflammatory injury [124]. In parallel, phospholipases, including phospholipase A_2_ (PLA_2_) and phospholipase D (PLD), contribute to host-cell membrane damage, with encephalitis-associated genotypes demonstrating increased enzymatic activity [125]. Neuraminidase production may further alter CNS glycolipid composition and contribute to neuronal vulnerability, although this mechanism remains less well characterized [126].

#### 3.3.4. Host Cell Death, Apoptosis and Dissemination

Host-cell death represents a final convergent mechanism linking tissue injury, barrier disruption, and dissemination. Programmed cell death pathways are activated during infection and further contribute to tissue destruction and loss of barrier integrity. Virulent genotypes, particularly T1-T4, can induce apoptosis in endothelial and neuronal cells through calcium dysregulation, Bax-dependent signaling, caspase activation, and proinflammatory cytokine release [71,127].

In addition to apoptosis, extracellular adenosine diphosphate (ADP) released by or generated during the activity of *Acanthamoeba* trophozoites may promote Ca^2+^ influx, membrane disruption, and calcium-dependent host-cell death. This mechanism may amplify local tissue injury and facilitate parasite dissemination by weakening cellular barriers [128,129]. Together, adhesion, phagocytosis, protease-mediated barrier disruption, phospholipase activity, and ADP-associated host-cell death provide a mechanistic basis for tissue invasion, CNS dissemination, and progression of GAE. These converging mechanisms allow *Acanthamoeba* spp. to transition from environmental persistence to invasive CNS infection, particularly in susceptible hosts.

### 3.4. Immune Response to Acanthamoeba spp.

The CNS is regulated by specialized immune mechanisms shaped by the BBB and the restricted access of peripheral immune cells. During CNS invasion by *Acanthamoeba* spp. and development of GAE, these features strongly influence the dynamics and effectiveness of host defense [7]. The first line of CNS defense is mediated by innate immunity, with microglia, the resident macrophage-like immune cells of neural tissue, playing a central role [130].

**Figure 2:**
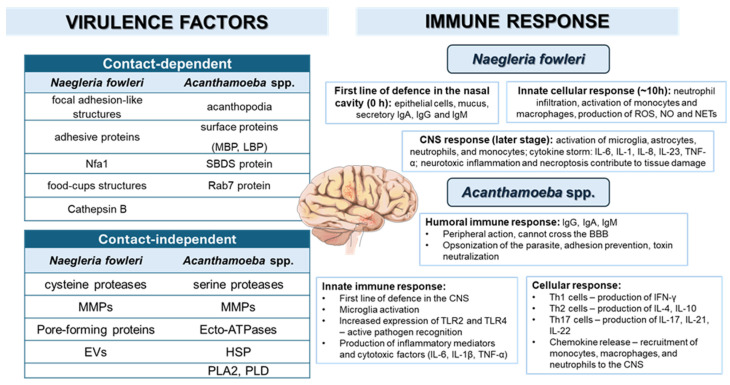
Comparative overview of virulence mechanisms, pathogenic pathways, and host immune responses involved in CNS infections caused by *Naegleria fowleri* and *Acanthamoeba* spp. (The figure was prepared by the authors using Microsoft PowerPoint LTSC MSO 2021 (Version 16.0.14334.20756, 64-bit; Microsoft Corporation). Brain image adapted from Servier Medical Art by Servier, CC BY 4.0.). **Abbreviations:** BBB, blood–brain barrier; CNS, central nervous system; Ecto-ATPases, ecto-adenosine triphosphatases; EVs, extracellular vesicles; h, hours; HSP, heat shock protein; IFN-γ, interferon gamma; IgA, immunoglobulin A; IgG, immunoglobulin G; IgM, immunoglobulin M; IL-1β, interleukin-1 beta; IL-4, interleukin-4; IL-6, interleukin-6; IL-8, interleukin-8; IL-10, interleukin-10; IL-17, interleukin-17; IL-21, interleukin-21; IL-22, interleukin-22; IL-23, interleukin-23; LBP, laminin-binding protein; MBP, mannose-binding protein; MMPs, matrix metalloproteinases; NETs, neutrophil extracellular traps; Nfa1, *Naegleria fowleri* adhesion protein 1; NO, nitric oxide; PLA2, phospholipase A2; PLD, phospholipase D; Rab7, Ras-related protein Rab-7; ROS, reactive oxygen species; SBDS protein, Shwachman–Bodian–Diamond syndrome protein; spp., species plural; Th1, T helper 1; Th2, T helper 2; Th17, T helper 17; TLR2, Toll-like receptor 2; TLR4, Toll-like receptor 4; TNF-α, tumor necrosis factor-alpha.

Importantly, neuroinflammation in GAE appears to be temporally heterogeneous and may remain limited during the earliest stages of CNS invasion. In an experimental intranasal model of *Acanthamoeba* genotype T4 infection, trophozoites crossed the nasal and olfactory epithelium through slow, contact-dependent migration between intercellular junctions, without overt cytolysis or prominent host inflammation. These findings suggest that early *Acanthamoeba* neuroinfection may be initially driven by mechanically mediated tissue penetration before a fully developed inflammatory response emerges [131]. As infection progresses and amoebae reach deeper CNS compartments, innate immune mechanisms become increasingly relevant. Microglia recognize pathogens through pattern recognition receptors, including Toll-like receptors (TLRs), leading to cellular activation and inflammatory signaling [8,132]. In murine infection models, increased expression of TLR2 and TLR4 has been detected in neural and vascular CNS-associated cells, indicating active pathogen recognition during infection [133]. Activated microglia can phagocytose *Acanthamoeba* trophozoites and release pro-inflammatory cytokines, including TNF-α, IL-1β, and IL-6 [132,134,135]. In vitro studies using *Acanthamoeba castellanii* genotype T4 have also shown induction of both pro-inflammatory cytokines and IL-10 in human monocytes and macrophages, suggesting simultaneous inflammatory activation and immune modulation [136]. Adaptive immune responses during CNS *Acanthamoeba* infection appear limited and predominantly cell-mediated. T helper 1 (Th1) responses, characterized by interferon gamma (IFN-γ), enhance microglial and macrophage activity and may amplify inflammatory signaling [86,137,138,139]. In immunosuppressed mice, Th1-skewed responses may contribute to greater tissue injury, whereas immunocompetent hosts also demonstrate T helper 2 (Th2) and T helper 17 (Th17) activity [86]. Th2-associated cytokines, including interleukin-4 (IL-4) and IL-10, may help limit excessive inflammation and protect neural tissue [140]. Th17 responses may be either protective or pathogenic depending on the cytokine profile and inflammatory context [141,142]. Interleukin-17 receptor (IL-17R)-mediated chemokine production by CNS-resident and endothelial cells promotes recruitment of monocytes, macrophages, neutrophils, and lymphocytes, supporting parasite clearance but potentially increasing BBB permeability and secondary inflammation [142,143]. Thus, balanced Th1/Th2/Th17 activity, potentially modulated by neurotrophic signaling, may be important for infection control while limiting CNS damage [144]. Humoral immunity appears to play a more limited role in cerebral acanthamoebiasis because antibody penetration across the BBB is restricted and antibodies are generally insufficient to eliminate amoebae within neural tissue [145]. Nevertheless, infection can stimulate IgG, IgM, and IgA responses that may neutralize trophozoites in blood or peripheral tissues, inhibit adhesion and motility, and reduce amoebic cytotoxicity [85,146,147]. Antibodies and complement may also promote lysis or opsonization in the presence of phagocytes, thereby supporting peripheral parasite clearance [65,148,149]. Overall, cellular immunity appears to be the principal determinant of CNS defense, whereas humoral responses may contribute mainly during early or peripheral stages of infection. Key virulence factors and host immune responses underlying the pathogenesis of PAM and GAE are summarized in Figure 2.

## 4. Diagnostic Strategies for Free-Living Amoebae Infections

**Figure 3:**
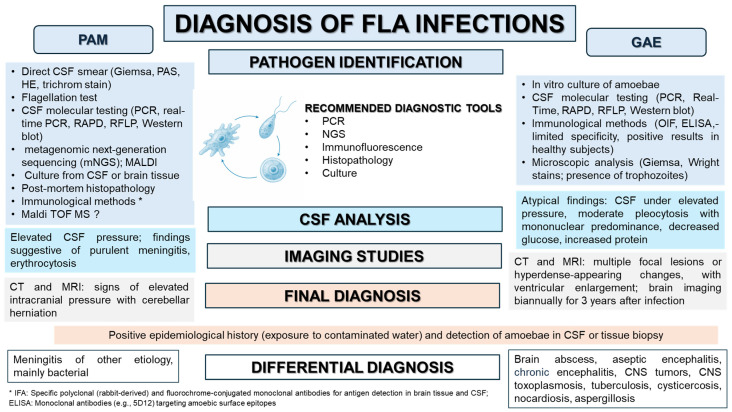
Overview of the diagnostic strategy for central nervous system infections caused by free-living amoebae. The schematic highlights differential diagnostic features of PAM and GAE and integrates clinical presentation, epidemiological exposure, laboratory findings, neuroimaging characteristics, and confirmatory molecular diagnostics. The figure was prepared by the authors using Microsoft PowerPoint LTSC MSO 2021 (Version 16.0.14334.20756, 64-bit; Microsoft Corporation). Only the graphical illustration was generated with the assistance of ChatGPT (OpenAI, https://chatgpt.com); the scientific content, labels, and interpretation were prepared and verified by the authors. **Abbreviations:** CNS, central nervous system; CSF, cerebrospinal fluid; CT, computed tomography; ELISA, enzyme-linked immunosorbent assay; FLA, free-living amoebae; GAE, granulomatous amoebic encephalitis; H&E, hematoxylin and eosin; MALDI-TOF MS, matrix-assisted laser desorption/ionization time-of-flight mass spectrometry; mNGS, metagenomic next-generation sequencing; MRI, magnetic resonance imaging; OIF, indirect immunofluorescence; PAM, primary amoebic meningoencephalitis; PAS, periodic acid–Schiff; PCR, polymerase chain reaction; RAPD, random amplified polymorphic DNA; RFLP, restriction fragment length polymorphism.

Diagnostic evaluation of FLA infections combines both conventional and advanced diagnostic approaches, such as microscopy, culture, PCR-based assays, direct immunofluorescence, and immunohistochemistry [22,40]. Given the high mortality associated with FLA infections, rapid and accurate diagnosis is essential for timely therapeutic intervention [150]. Figure 3 outlines the proposed diagnostic algorithm.

In clinical practice, initial assessment typically relies on microscopy and culture of biopsy specimens and cerebrospinal fluid, increasingly complemented by molecular diagnostic techniques [15]. Conventional and real-time PCR methods provide high sensitivity and specificity, whereas multiplex PCR enables rapid simultaneous detection of multiple pathogens, thereby improving diagnostic efficiency and clinical decision-making [40,151]. Despite advances in molecular diagnostics, early detection of FLA infections remains challenging due to nonspecific clinical manifestations and limited clinical awareness. These limitations highlight the need for improved rapid diagnostic approaches.

## 5. Treatment

Treatment of central nervous system infections caused by FLA remains highly challenging, as no uniformly effective therapy has been established. Current management relies largely on multidrug combinations derived from limited clinical guidelines and case reports [152,153]. Delayed diagnosis, poor BBB penetration, systemic toxicity, and the limited in vivo efficacy of compounds demonstrating activity only in vitro contribute to mortality rates approaching 80–98% [23,154]. These limitations highlight the urgent need for novel therapeutic strategies and improved drug delivery systems capable of overcoming BBB-related pharmacological barriers. An important determinant of therapeutic efficacy in FLA infections is the developmental stage of the amoeba. Trophozoites are metabolically active, invasive forms responsible for tissue destruction and are generally more susceptible to pharmacological treatment. In contrast, cysts are dormant or low-metabolic forms protected by a resistant wall, which limits drug penetration and increases tolerance to anti-amoebic agents [155]. This distinction is clinically important in *Acanthamoeba* spp. infections, where both trophozoites and cysts may contribute to persistence and relapse. By contrast, *N. fowleri* does not form cysts in human tissue; therefore, PAM therapy is directed primarily against trophozoites [9]. For GAE, an ideal regimen should target both trophozoites and cysts; however, no standardized stage-specific treatment strategy currently exists.

Therapeutic approaches can be broadly categorized into (i) conventional regimens based on the Centers for Disease Control and Prevention (CDC) recommendations; (ii) therapeutic combinations and surgical interventions associated with reported survival; and (iii) emerging and experimental therapies, including repurposed drugs, metal-based systems, drug conjugates, and phytochemical-enhanced strategies.

### 5.1. Conventional Regimens (CDC-Based Therapy)

Current treatment of PAM and GAE relies on aggressive multidrug combinations recommended by CDC [23,152,156,157,158]. For PAM, CDC-recommended regimens typically include amphotericin B; azithromycin; an azole antifungal, such as fluconazole or posaconazole; rifampicin; miltefosine; and adjunctive dexamethasone to mitigate cerebral inflammation and reduce intracranial pressure [156]. Therapeutic hypothermia and aggressive management of cerebral edema have also been used in selected survivors as supportive interventions [159]. For GAE, CDC-recommended multidrug therapy includes pentamidine; sulfadiazine; flucytosine; miltefosine; and an azole antifungal, most commonly fluconazole or voriconazole [23,152,157,158]. Additional agents, including amphotericin B, metronidazole, trimethoprim–sulfamethoxazole, rifampicin, ethambutol, acyclovir, or corticosteroids, have been used in selected cases depending on disease severity, host immune status, drug availability, and clinical response [152,157]. Despite aggressive multidrug therapy, outcomes remain poor, highlighting the urgent need for novel therapeutic strategies, improved CNS drug delivery approaches, and early diagnostic intervention.

### 5.2. Reported Survival Outcomes and Survival-Associated Therapeutic Combinations

Survival from PAM remains exceedingly rare. Documented survivors have generally been treated with early, aggressive multidrug therapy, most commonly including amphotericin B, azithromycin, fluconazole, rifampicin, miltefosine, and adjunctive dexamethasone [156]. Laboratory-confirmed survival cases are summarized in Table 2. Although approximately 32 PAM survivors have been described in the broader literature, only nine cases fulfilled laboratory criteria confirming *N. fowleri* infection. In the remaining reports, the causative organism was not definitively confirmed, and diagnosis relied largely on indirect or morphological evidence [160,161,162]. This distinction is important because infections caused by other FLA cannot be excluded, particularly in regions with limited access to confirmatory molecular diagnostics [162]. Across confirmed survival cases, favorable outcomes appear to be associated with early recognition, rapid initiation of combination therapy, aggressive management of intracranial complications, and access to miltefosine-containing regimens when available. In GAE, survival is also uncommon. By 2020, only 19 survival cases had been reported among immunocompetent and immunosuppressed patients [96,108,163]. Reported favorable outcomes were associated with multidrug therapy, including combinations of miltefosine, metronidazole, rifampicin, azithromycin, pentamidine isethionate, and co-trimoxazole. In selected immunocompetent patients, surgical excision or debulking of focal abscess-like lesions combined with pharmacological therapy, particularly miltefosine-based regimens, was associated with survival [76,96,108,163]. By contrast, fatal outcomes were frequently reported in patients receiving less intensive regimens, such as fluconazole and co-trimoxazole alone [75]. Collectively, reported survival cases suggest that favorable outcomes in PAM and GAE are associated with early diagnosis; rapid initiation of multidrug therapy; effective control of intracranial complications; and, in selected GAE cases, surgical excision or debulking of focal lesions. However, these observations are derived mainly from case reports and small case series and should therefore be interpreted as survival-associated patterns rather than evidence-based standardized treatment regimens.

### 5.3. Emerging and Experimental Therapeutic Strategies

Several emerging compounds and delivery platforms have demonstrated anti-amoebic activity and may improve future therapeutic outcomes when incorporated into rational combination regimens. However, at present, most evidence remains preclinical; therefore, these approaches should be interpreted as experimental strategies rather than established treatment options. Novel drug delivery strategies are also being explored to improve CNS penetration and reduce systemic toxicity. Intranasal administration of amphotericin B and dexamethasone using automated nebulization systems may enable controlled delivery along the olfactory route, potentially bypassing the BBB and increasing drug accumulation in the olfactory bulb and frontal cortex [25,170]. Although still experimental, this approach directly addresses two major therapeutic barriers in PAM: delayed CNS drug exposure and dose-limiting systemic toxicity.

#### 5.3.1. Repurposed Drugs

Drug repurposing represents an attractive strategy because several approved compounds have well-characterized pharmacological profiles and may be rapidly redirected toward rare CNS infections. Nitroxoline has demonstrated potent in vitro activity against both *N. fowleri* and *Acanthamoeba* spp. and has been evaluated within Investigational New Drug (IND) protocols [156,157,171,172]. Auranofin, a gold-containing antirheumatic drug, exhibits inhibitory activity against both *Acanthamoeba* spp. and *N. fowleri* and has attracted interest because of its reported BBB penetration [173,174]. Other repurposed agents, including statins, prochlorperazine-based combinations, digoxin, and amlodipine, have demonstrated amoebicidal activity in vitro through disruption of cellular homeostasis or apoptosis-like mechanisms [175,176]. Although these agents have not yet been shown to improve survival in clinical settings, their pharmacological properties and anti-amoebic activity support further evaluation as components of future combination regimens.

#### 5.3.2. Stage-Specific Anti-Amoebic Biocides, Diamidines and Photodynamic Therapy

Several established anti-amoebic agents used primarily for the treatment of *Acanthamoeba keratitis* have provided important experimental data on stage-specific susceptibility. Biguanides, particularly polyhexamethylene biguanide (PHMB) and chlorhexidine, as well as diamidines including propamidine, hexamidine, dibromopropamidine, and pentamidine, have shown activity against *Acanthamoeba* trophozoites and cysts in vitro [177,178]. This distinction is clinically relevant because cysts may survive therapy, subsequently excyst, and contribute to infection recurrence. However, the efficacy of some agents, including miltefosine and selected diamidines, appears variable depending on assay conditions and the developmental stage tested [177]. Photodynamic therapy, including photodynamic therapy based on chlorin e6 (chlorin e6-based PDT), has also demonstrated dose-dependent amoebicidal effects against both trophozoites and cysts, although this approach remains experimental and its applicability to CNS disease is uncertain [178]. Together, these findings highlight the need to evaluate candidate therapies against both developmental stages rather than trophozoites alone.

#### 5.3.3. Nanotechnology-Based Systems: Metal-Based, Drug-Conjugate and Phytochemical-Enhanced Approaches

Nanotechnology-based systems are being explored to improve drug stability, bioavailability, CNS delivery, and host-cell selectivity in amoebic infections [179,180]. These approaches can be grouped into three main categories. First, metal and metal oxide nanoparticles (NPs), including zinc oxide nanoparticles (ZnO NPs), silver nanoparticles (AgNPs), and gold nanoparticles (AuNPs), exhibit intrinsic anti-amoebic activity and may exert their effects through membrane disruption and oxidative stress-related mechanisms [181,182,183,184,185,186]. Second, drug–nanoparticle conjugates, such as ZnO–β-cyclodextrin–ceftriaxone systems, have been shown to reduce the growth of *Acanthamoeba castellanii*, cytopathogenicity, and encystation with limited host-cell toxicity [187]. Related ZnO-based conjugates have also demonstrated enhanced activity against *N. fowleri* while preserving host-cell viability [181]. Third, phytochemical-enhanced nanoparticle systems, including nanoparticles combined with curcumin, terpenes, or other plant-derived compounds, may increase anti-amoebic efficacy, including activity against cyst stages, while reducing toxicity [25,182,183,184]. Although these platforms remain preclinical, they may help address two major barriers to survival: inadequate CNS drug delivery and systemic toxicity.

#### 5.3.4. Plant-Derived Compounds and Immunological Strategies

Plant-derived compounds represent an additional experimental therapeutic avenue. Flavonoids, terpenoids, and other secondary metabolites, including demethoxycurcumin, kaempferol, resveratrol, betulinic acid, and extracts from *Pinus densiflora*, *Artemisia annua*, and other plants, have demonstrated amoebicidal activity against *N. fowleri* and *Acanthamoeba* spp. in vitro [188,189,190,191,192,193]. These compounds may reduce trophozoite viability, interfere with encystation as well as adhesion, and decrease cytopathogenicity while maintaining relatively low host-cell toxicity. However, their clinical translation requires standardized extract characterization, pharmacokinetic evaluation, toxicity assessment, and validation in relevant in vivo CNS infection models.

Immunological strategies, including multi-epitope vaccine candidates against *N. fowleri*, have also been proposed using in silico approaches [194,195]. These platforms aim to induce humoral and cellular immune responses; however, they remain at an early stage and require experimental validation. Overall, emerging therapies may improve future survival by enhancing CNS delivery, reducing systemic toxicity, targeting cyst persistence, and expanding the range of drugs available for rational combination therapy. At present, however, most approaches remain preclinical and should not be regarded as substitutes for rapid diagnosis, aggressive multidrug treatment, and effective management of intracranial complications.

## 6. Conclusions and Future Directions: A Translational Roadmap

**Figure 4:**
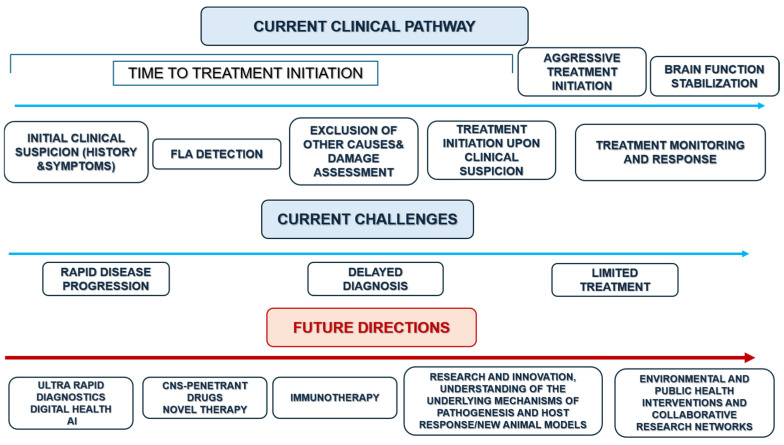
Proposed translational roadmap for improving outcomes in PAM and GAE, illustrating key milestones, current limitations, and future priorities in diagnostics, therapy development, environmental surveillance, One Health strategies, and international collaborative research.

Free-living amoebae, particularly *N. fowleri* and *Acanthamoeba* spp., remain among the most devastating causes of rare central nervous system infections, with persistently high mortality driven by rapid disease progression, delayed diagnosis, and limited therapeutic efficacy. Despite advances in molecular diagnostics, pathogen biology, and experimental therapies, substantial improvements in patient outcomes will require a shift from reactive management toward proactive strategies based on early recognition, rapid initiation of combination therapy, and improved understanding of host–pathogen interactions. Future progress should prioritize ultra-rapid diagnostic platforms, novel BBB-penetrating therapies, host-directed and immunomodulatory approaches, and more predictive experimental models, supported by enhanced environmental surveillance and global clinical awareness within the One Health framework. Given that PAM and GAE are rare but frequently fatal diseases, progress will require coordinated international collaboration, shared clinical registries, harmonized diagnostic workflows, and standardized treatment reporting. As illustrated in Figure 4, an integrated translational roadmap linking diagnostics, therapeutic innovation, surveillance, and collaborative research is urgently needed to translate emerging scientific advances into improved survival.

## Figures and Tables

**Table 1:** Species-dependent clinical manifestations of neuroinvasive free-living amoebae infections.

Disease Entity	Primary Amoebic Meningoencephalitis	Granulomatous Amoebic Encephalitis
Feature	*Naegleria fowleri*	*Acanthamoeba* spp.
Typical host profile	Usually previously healthy immunocompetent individuals, often children or young adults with freshwater exposure	Mainly immunocompromised patients; occasional cases in immunocompetent hosts have also been reported
Disease course	Short incubation period followed by acute symptom onset and fulminant neurological progression	Insidious onset with subacute or chronic progression
Dominant neurological presentation	Meningoencephalitis-like illness with fever, severe headache, neck stiffness, seizures, altered mental status, and coma	Nonspecific neurological symptoms, seizures, focal neurological deficits, altered mental status, ataxia, aphasia, behavioral changes, or coma
Neuroimaging/clinical mimicry	Often resembles acute bacterial meningitis	May mimic tumor, abscess, stroke, lymphoma, or other opportunistic CNS infections
Predominant neuropathology	Acute hemorrhagic and necrotizing meningoencephalitis, often involving the olfactory bulbs and frontal lobes	Granulomatous and necrotizing encephalitis, vasculitis, abscess-like lesions, and perivascular amoebae
Type of inflammation	neutrophilic	granulomatous
Key diagnostic challenge	Rapid progression leaves limited time for diagnosis and treatment	Nonspecific and slowly progressive presentation often delays recognition
Therapeutic implication	Treatment mainly targets trophozoites, as cysts are not formed in human brain tissue	Therapy should ideally target both trophozoites and drug-resistant cysts

**Table 2:** Laboratory-confirmed survival cases of primary amoebic meningoencephalitis (PAM) caused by *Naegleria fowleri*. The table presents patient demographics, exposure history, treatment strategies, and clinical reports documenting successful outcomes.

Case Report	Country of Exposure	Year	Age/Sex	Water-Related Activities	Treatment	Reference
1	Australia	1971	14 y; M	-	Amphotericin B	[164]
2	USA	1978	9 y; F	Bathing in hot springs	Amphotericin B, miconazole, rifampicin; dexamethasone and phenytoin (symptomatic)	[165]
3	Mexico	2003	10 y; M	Swimming in an irrigation canal	Amphotericin B, fluconazole, rifampicin, dexamethasone	[166]
4	USA	2013	12 y; F	Swimming in an outdoor water park	Amphotericin B, fluconazole, rifampicin, azithromycin, miltefosine, dexamethasone	[159]
5	USA	2013	8 y; M	Playing on the riverbank	Amphotericin B, fluconazole, rifampicin, azithromycin, miltefosine, dexamethasone	[167]
6	Pakistan	2015	25 y; M	Swimming in a river	Amphotericin B, fluconazole, azithromycin, miltefosine, rifampicin, chlorpromazine	[168]
7	USA	2016	16 y; M	Swimming in a freshwater water park	Amphotericin B, fluconazole, azithromycin, miltefosine, rifampicin, dexamethasone	[169]
8	Pakistan	2023	22 y; M	-	Amphotericin B, fluconazole, azithromycin, miltefosine, rifampicin	[150]
9	India	2024	23 y; M	swimming and bathing in a pond	Amphotericin B, rifampicin, fluconazole, miltefosine, azithromycin	[160]

Abbreviations: F, female; M, male.

## Data Availability

No new data were created or analyzed in this study. Data sharing is not applicable to this article.

## Abbreviations

The following abbreviations are used in this manuscript:

ADP	adenosine diphosphate
BBB	blood–brain barrier
CDC	Centers for Disease Control and Prevention
CNS	central nervous system
CSF	cerebrospinal fluid
ECM	extracellular matrix
EGFR	epidermal growth factor receptor
EVs	extracellular vesicles
FLA	free-living amoeba
GAE	granulomatous amoebic encephalitis
HSP70	heat shock protein 70
IFN-γ	interferon gamma
IL	interleukin
LBP	laminin-binding protein
MALDI-TOF MS	matrix-assisted laser desorption/ionization time-of-flight mass spectrometry
MBP	mannose-binding protein
MMPs	matrix metalloproteinases
mNGS	metagenomic next-generation sequencing
NETs	neutrophil extracellular traps
Nfa1	*Naegleria fowleri* adhesion protein 1
NO	nitric oxide
OIF	indirect immunofluorescence
PAM	primary amoebic meningoencephalitis
PI3K	phosphatidylinositol 3-kinase
PLA2	phospholipase A2
PLD	phospholipase D
RAPD	random amplified polymorphic DNA
RFLP	restriction fragment length polymorphism
ROS	reactive oxygen species
Th	T helper cell
TLRs	Toll-like receptors
TNF-α	tumor necrosis factor alpha
ZO-1	zonula occludens-1
